# Optimized Conformal Total Body Irradiation methods with Helical TomoTherapy and Elekta VMAT: Implementation, Imaging, Planning and Dose Delivery for Pediatric Patients

**DOI:** 10.3389/fonc.2022.785917

**Published:** 2022-03-10

**Authors:** Anna Anzorovna Loginova, Diana Anatolievna Tovmasian, Anastasiya Olegovna Lisovskaya, Daria Alexeevna Kobyzeva, Michael Alexandrovich Maschan, Alexander Petrovich Chernyaev, Oleg Borisovich Egorov, Alexey Vladimirovich Nechesnyuk

**Affiliations:** ^1^ Dmitry Rogachev National Research Center of Pediatric Hematology, Oncology and Immunology, Moscow, Russia; ^2^ Faculty of Physics, Federal State Budget Educational Institution of Higher Education, M.V. Lomonosov Moscow State University, Moscow, Russia; ^3^ Research and Development Department, E&H Scientific, West Richland, WA, United States

**Keywords:** pediatric, TBI, TMLI, VMAT, TomoTherapy, dose delivery, robustness, standardization

## Abstract

Optimized conformal total body irradiation (OC-TBI) is a highly conformal image guided method for irradiating the whole human body while sparing the selected organs at risk (OARs) (lungs, kidneys, lens). This study investigated the safety and feasibility of pediatric OC-TBI with the helical TomoTherapy (TomoTherapy) and volumetric modulated arc (VMAT) modalities and their implementation in routine clinical practice. This is the first study comparing the TomoTherapy and VMAT modalities in terms of treatment planning, dose delivery accuracy, and toxicity for OC-TBI in a single-center setting. The OC-TBI method with standardized dosimetric criteria was developed and implemented with TomoTherapy. The same OC-TBI approach was applied for VMAT. Standardized treatment steps, namely, positioning and immobilization, contouring, treatment planning strategy, plan evaluation, quality assurance, visualization and treatment delivery procedure were implemented for 157 patients treated with TomoTherapy and 52 patients treated with VMAT. Both modalities showed acceptable quality of the planned target volume dose coverage with simultaneous OARs sparing. The homogeneity of target irradiation was superior for TomoTherapy. Overall assessment of the OC-TBI dose delivery was performed for 30 patients treated with VMAT and 30 patients treated with TomoTherapy. The planned and delivered (sum of doses for all fractions) doses were compared for the two modalities in groups of patients with different heights. The near maximum dose values of the lungs and kidneys showed the most significant variation between the planned and delivered doses for both modalities. Differences in the patient size did not result in statistically significant differences for most of the investigated parameters in either the TomoTherapy or VMAT modality. TomoTherapy-based OC-TBI showed lower variations between planned and delivered doses, was less time-consuming and was easier to implement in routine practice than VMAT. We did not observe significant differences in acute and subacute toxicity between TomoTherapy and VMAT groups. The late toxicity from kidneys and lungs was not found during the 2.3 years follow up period. The study demonstrates that both modalities are feasible, safe and show acceptable toxicity. The standardized approaches allowed us to implement pediatric OC-TBI in routine clinical practice.

## Introduction

Total body irradiation (TBI) is used in the treatment of hematological malignancies as part of conditioning regimens before hematopoietic stem cell transplantation (HSCT). Conventional TBI at extended source-surface distances has been established and demonstrated to be a reliable method, but its use is limited by its high toxicity (1–3).

There are methods of optimized TBI with a relatively short source-to-surface distance and intensity modulation (4, 5). They make it possible to use more homogeneous targeted irradiation than conventional TBI by reducing the dose to the organs at risk (OARs), but such methods are still not considered conformal.

The first optimized conformal TBI and total marrow irradiation (TMI) methods were tested using helical TomoTherapy (6–8) and later with a standard linac (9, 10). A benefit with regard to dose distribution and selective OAR dose sparing was demonstrated by other authors in an adult cohort (11–13) and by Gruen et al. in a pediatric cohort (14).

Currently, much attention is given to total marrow irradiation (TMI) and total marrow and lymphoid irradiation (TMLI) (15). TMI and TMLI show promise with respect to the toxicity profile because targeted irradiation enables reduction of the dose to the OARs and the feasibility of possible dose escalation to improve disease control in refractory and relapsed patients (15–17).

Potentially, TMI and TMLI could increase the relapse rate caused by underdosages to nontargeted regions. Kim et al. investigated extramedullary relapse in adult patients treated with TMLI and did not find an association of its incidence with lower dose regions (18). However, the application of TMI and TMLI as standard treatment approaches for pediatric patients with leukemia requires additional research to ensure safety, quality, clinical outcomes, toxicity, and feasibility.

Pediatric TBI approaches are quite different among clinics and depend on technical capacities and individual establishment (19, 20). There are no common practical recommendations for OC-TBI treatment planning and preparation. The main task for our department was to develop an optimized conformal total body irradiation (OC-TBI) method with sparing of the selected organs at risk (OARs) (lungs, kidneys, and lenses) and implement it in clinical practice. The development and implementation of the TomoTherapy-based OC-TBI method was carried out by our Center between 2014 and 2017. The rationale was to provide OC-TBI as close as possible to conventional TBI, which is used as standard treatment of care and assumes impartial lung shielding with better outcomes at lung doses <8 Gy (19–21). To achieve this we prescribed a minimum dose of 6 Gy to lungs. There is no direct clinical evidence of the minimum dose requirement for TBI treatment, but studies have shown that fractionated conventional TBI <9–10 Gy results in increased nonengraftment and disease relapse (22, 23). Prescription of a minimum dose up to 9–10 Gy would negatively affect lung and kidney sparing in treatment plan optimization.

Furthermore, the same approach has been applied for VMAT (since 2017). One of the important tasks was to provide the possibility to perform similar OC-TBI treatment for our patients using both modalities. This is the first study comparing the TomoTherapy and VMAT modalities in terms of treatment planning, dose delivery accuracy, and safety for OC-TBI in a single-center setting. The results of this study can be useful for clinics considering the possibility of implementing OC-TBI on an ongoing basis, given the different equipment used for this purpose and a detailed description of the methods.

## Materials and Methods

### Patient Characteristics

Between July 2014 and July 2021, OC-TBI was implemented for a total of 341 pediatric patients who received HSCT, 279 of whom underwent helical TomoTherapy Hi-Art system (Accuray Inc., Sunnyvale, CA, USA) and 62 of whom underwent Elekta VMAT. Standardized treatment steps, namely, positioning and immobilization, contouring, treatment planning strategy, plan evaluation, quality assurance, visualization and treatment delivery procedure, were implemented for 157 patients treated with TomoTherapy and 52 patients treated with VMAT from June 2017 to May 2021. Patient age in the standardized group varied from 3 to 21 years (median—10.4 years old). Twenty patients were treated under general anesthesia. Anesthesia was delivered according to age. We also used general anesthesia in some cases with unsatisfactory patient psychological and performance status. The median age of patients receiving TBI under general anesthesia was 4.8 years.

We followed up patients for acute toxicity (nausea/vomiting/diarrhea, headache) during radiation therapy, subacute toxicity (IP) up to the 100th day after HSCT and late toxicity in the lungs and kidneys for at least 100 days after HSCT in accordance with the RTOG/EORTC scale (24).

### Standardized Immobilization and CT Simulation

Standard CT imaging preceded whole body immobilization in the supine position using a vacuum mattress with rigid attachments to the couch with head fixation using pillows and thermoplastic masks (Elekta, UK, Crawley). The hands and arms of the patient were placed as close as possible to the body to minimize the lateral distance and improve the target dose homogeneity for the TomoTherapy plan with helical delivery and to maximize the body volume within the field of view of the Megavoltage Computed Tomography (MVCT) or Cone Beam Computed Tomography (CBCT) imaging. The body of the patient was set up tightly into the mattress, and the feet rested firmly on the mattress as described by Haraldsson et al. (25). Longitudinal laser lines were marked along the entire body to facilitate reproducibility of the patient setup.

CT images were obtained using a LightSpeed RT16 Computer Tomography (General Electric, Boston, USA) scanner. Images were acquired in free breathing with a slice thickness of 5 mm using 120 kV X-ray tube voltage and the largest available field of view (FOV) of 65 cm.

Images for patients with heights greater than 115 cm were obtained using two scans carried out in opposite directions to overcome the limitations of the treatment length for both the TomoTherapy and Elekta treatment units. The first scan included the upper body up to the knees in the head-first supine position of the patients. The second scan started from the tips of the feet to the pelvis in a feet-first supine position using a vacuum mattress rotated by 180°. Scan overlap was needed to perform image registration and manage junction dose. The fiducial junction markers were placed on the midsection of the patient to enable control of the junction area.

### Dose Prescription

A dose of 12 Gy was given twice daily in 6 fractions or once daily in 4 fractions as part of the HSCT conditioning regimens. The planned target volume (PTV) included the whole body with a 3-mm inside margin and excluded the lungs, kidneys, and lenses.

The dose to the lung was prescribed at V8 <40% (that is, the volume of each lung receiving 8 Gy was not to exceed 40% of the whole lung volume) with a minimum dose of at least 6 Gy. The mean kidney dose was prescribed at <8 Gy.

Dose constraints to the eye lens were not prescribed, but effort was made to reduce the dose to these organs while maintaining the coverage of 95% of the prescribed dose in the adjacent PTV. For this aim, the PTV area near the eye was additionally contoured and used as a separate target when optimizing the plan.

### Treatment Plan Calculation Strategy

#### TomoTherapy Optimized Conformal Total Body Irradiation Calculation Strategy

##### General OC-TBI Treatment Planning Strategy for TomoTherapy

OC-TBI plans for TomoTherapy were created using the non-Volo TomoTherapy 4.5 (Accuray Inc., Sunnyvale, CA, USA) treatment planning system (TPS) and treatment planning software using Helical dose delivery without the TomoEdge option. In the OC-TBI method, the OARs underwent dose reduction and minimum dose prescription simultaneously. Even though the lungs and kidneys were not included in the PTV, they were nonetheless used as part of the target during the dose optimization process.

##### Patient Size-Dependent OC-TBI Treatment Planning Strategy for TomoTherapy

Helical TomoTherapy delivery is associated with peripheral dose heterogeneity, which depends on plan modulation (26, 27). To reduce this effect, we chose TomoTherapy plan parameters depending on the size of the patient. For patients with height <115 cm, we used a field width of 2.5 cm, pitch of 0.43 and modulation factor of 1.9. For patients with height >115 cm and with right to left PTV size <17 cm, we used a field width of 5 cm, pitch of 0.43, and modulation factor of 1.9. For patients with height >115 cm and right to left PTV size >17 cm, we used a field width of 5 cm, pitch = 0.287 and modulation factor = 2.6.

##### Junction Between Upper and Lower Body Management for TomoTherapy-Based OC-TBI Treatment Planning

**Figure 1:**
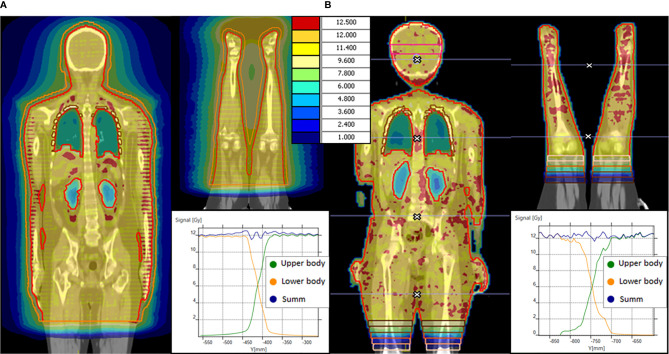
OC-TBI dose distribution and dose profile in junction area between upper and lower body for **(А)** TomoTherapy and **(B)** VMAT.

For patients with height >115 cm separate series of images were acquired for the upper and lower bodies, dose calculations were carried out independently for the two series (**Figure 1A**).

Our version of the TomoTherapy TPS does not allow image fusion and dose optimization of the lower body based on the upper body dose distribution. There is no possibility to compensate for possible deviations of the upper-body junction from the prescribed gradient while optimizing the lower body dose. The optimization of the junction area therefore would require ensuring a precisely prescribed dose gradient. For this reason, we use a nonoptimized method with an offset between the PTVs (28, 29).

In the case of TomoTherapy, the dose decrease at the edge of the field in the longitudinal direction was found to be quite smooth due to helical dose delivery (**Figure 1 A** **)**.

During plan optimization, a contour offset was maintained between the upper and lower PTVs, while the dose distribution in the junction area was set to satisfy a uniform 90–120% of the prescribed dose. The dimension of the offset depends on the field width, pitch, modulation factor, optimization of the selected plan and CT slice thickness; our typical value is 5.5 cm. The external software MIM Maestro™ (MIM Software Inc., Cleveland, OH, USA) with the image fusion option was used to ensure that the dose in the junction area was maintained within the prescribed dose.

##### Additional Property Providing TomoTherapy Plan Robustness to Possible Patient Positioning Errors

The concept of a virtual bolus was used to ensure that the treatment plan was less sensitive to patient positioning errors. To accomplish this, we created an additional structure, PTV + 1 cm, and optimized it as the target. Since the PTV was created using a negative 3 mm margin from the skin surface, the virtual bolus involved in the optimization was a shell around the PTV, consisting of a 3 mm thick skin layer and a 7 mm thick air layer. Fifty percent of the structure volume of PTV + 1 cm was prescribed a dose of 6 Gy. The prescription of a half dose to the virtual bolus allowed the optimizer to not create an excess fluence but one closer to that generated the PTV.

#### VMAT OC-TBI Calculation Strategy

##### General Treatment Planning Strategy for VMAT-Based OC-TBI

All VMAT-based plans were created using the multi-isocenter technique (30–34). Treatment plan optimization was performed using Monaco 5.11 treatment planning software (Elekta Inc., UK, Crawley) and the Monte Carlo algorithm with a statistical uncertainty of 3% per plan. A voxel size of 5 mm was chosen for the optimization stage. The final dose was recalculated with a 3 mm voxel size and statistical uncertainty of 1%.

Simultaneous optimization of all beams was carried out only for smaller patients, as presented in **Table 1**. In cases of taller patients, the PTV was split into several subsections and calculated one by one using the bias dose option.

**Table 1:** Patient size-dependent treatment planning strategies for VMAT-based OC-TBI.

Height of the patient, cm	Head	Chest	Abdomen	Pelvis	Upper legs	Lower legs	Number of isocenters
<105	One plan for whole body						4
	VMAT	VMAT	VMAT		VMAT		
105–145	upper body Plan				lower body Plan		6
	VMAT	VMAT	VMAT	VMAT	VMAT	VMAT	
>145	upper body Plan				lower-body plan		9
	VMAT	VMAT	VMAT	2 IMRT fields (10 and 170 Gantry angles) with couch rotated to 90°	2 AP-PA fields IMRT	2 AP-PA fields IMRT	

We used coplanar 360° VMAT dose delivery on an Elekta Synergy treatment unit equipped with an Agility collimator (Elekta Inc., UK, Crawley). The gantry was moved clockwise and counterclockwise, with positions of the isocenters differing from each other only in the longitudinal coordinate. The energy of the photon beams was set to 6 and 10 MeV. The collimator position was set to 90°, as described by Nalichowski et al. (35). The collimator jaws were selected in accordance with the individual anatomy of the patient to ensure coverage of the subregions of the corresponding patient, consisting of the areas of the head and neck, lungs, abdomen, and pelvis as described by Mancosu et al. (36). When irradiating the lower extremities, we used a static position for the gantry and several beams with intensity modulation and 90° rotation of the treatment couch.

##### Treatment Planning Strategy in Relation to Patient Size for VMAT-Based OC-TBI

To obtain a desired dose distribution, we developed three different treatment planning strategies depending on patient height. We used anteroposterior–posteroanterior IMRT beams for the pelvis and lower extremities for large patients, which significantly helped reduce the MU/fraction ratio and decrease the fraction time. The VMAT-based OC-TBI treatment planning strategies are presented in **Table 1**.

##### Management of Junction Between Upper and Lower Body for VMAT-Based OC-TBI

For the VMAT plan, we use gradient junction optimization described previously (37). In the junction area of the upper body, the PTV was divided into 5 sequential volumes (thickness 2 cm) with dose value prescriptions of 11, 9, 6, 3, and 1 Gy in the crania-caudal direction. Following rigid image registration, identical structures were created for the lower-body image series. Next, the bias dose option was used to prescribe dose values of 1, 3, 6, 9, and 11 Gy and obtain a total dose of 12 Gy for each sequential volume (**Figure 1B**).

##### Additional Properties Providing VMAT Plan Robustness to Possible Patient Positioning Errors

The treatment beams were positioned to cover the whole PTV with overlap along the longitudinal axis from 2 to 4 cm at the isocenter level, thereby providing the ability to automatically optimize the dose to the PTV, namely, the overlapped areas. Those areas were selected in a manner to eliminate or minimize intersection with the organs at risk.

To increase the plan robustness in relation to patient positioning, we used the Monaco 5.11 (Elekta Inc., UK, Crawley) Auto-Flash option with a 1 cm margin. After applying the Auto-Flash Option, an extension of the dose outside the body surface is created, so even under inhalation motion, irradiation of the superficial tissues can still be guaranteed (38).

### Standard Criteria for the Treatment Plan Evaluation and Comparison of OC-TBI Using TomoTherapy and VMAT

For the PTV and OARs, we established target dose values that we attempted to meet during the plan optimization step with the TomoTherapy treatment planning station (see **Table 2**). However, the plans were considered acceptable in terms of the deviation from the target values, which were defined as acceptable values for balancing planning time and plan complexity (**Table 2**).

**Table 2:** Acceptance criteria of OC-TBI plans for TomoTherapy.

Structure	Target value	Acceptable value
PTV	Mean dose (12 Gy) ± 2%	Mean dose (12 Gy) ± 5%
	D_98%_ >11.4 Gy	D_95%_ >11.4 Gy
	D_2%_ <13 Gy	D_5%_ <13 Gy
Ribs	D_95%_ >10 Gy	D_90%_ >10 Gy
Lung R,	V6 >99%	V6 >90%
Lung L	V8 <40%	V8 <40%
Kidney R, Kidney L	Dmean < 8 Gy	

The majority of patients were treated using TomoTherapy. To ensure consistent results, the same plan acceptance criteria (**Table 2**) were applied for the VMAT plans.

Standardized treatment entailing standardized positioning/immobilization, contouring, treatment planning strategies, plan evaluation, quality assurance, visualization and treatment delivery procedures was implemented for 157 patients treated with TomoTherapy and 52 patients treated with VMAT. The comparison of planned doses for the above two groups of patients was performed using the metrics presented in **Table 2** as the mean ± s.d.

The two modalities were compared using the mean dose (D mean) to the OARs, the near maximum dose D_2max_ and near minimum D_98min_ dose to the whole body PTV and Ribs volumes and the homogeneity index (HI), which describes the degree of uniformity of the target irradiation:

$HI=\frac{D_{2\max}-D_{98\min}}{D_{50\%}}\cdot 100\%$ , where D_50%_ is the median of the absorbed dose.

### Individual Quality Assurance Procedures for OC-TBI Treatment Plans

The individual quality assurance procedures included dosimetry checks for each treatment plan.

For the OC-TBI treatment plans, absolute dose measurements were performed using ionization chambers (ExtraDIN Chambers, A1SL), an 8-channel electrometer (TomoElectrometer) and a tissue-equivalent phantom (Cheese Phantom) provided by Accuray Inc. (Sunnyvale, CA, USA). Measurements were carried out by placing the ionization chambers at four selected locations: two corresponding to the OARs, and the other two to the target area. The maximum permissible deviation of the measured dose from the calculated dose was less than 3%. In-house software was used for additional quality assurance. Exit detector data from the onboard MVCT imaging system were obtained during the Static Couch quality assurance procedure, a procedure in which irradiation is performed in the absence of a phantom and movement of the couch while maintaining the movement of the gantry and collimator. The received signal is recorded by onboard detectors. Obtained data was compared with a sinogram from the TPS (39). The 2D-gamma index was used for data comparison of the selected treatment plan (40).

For the VMAT-based plans, individual quality assurance procedures included composite measurements (41) of the two-dimensional dose distributions using an array of MatriXX Evolution ionization chambers (IBA Dosimetry, Belgium) with applied angular correction (42).

The 3%/3 mm Gamma criterion was assessed with a 95% passing rate for both modalities.

### Pretreatment Visualization With MVCT and CBCT

Imaging procedures based on MVCT or CBCT were performed before each treatment session to verify the position of the patient. A single long MVCT scan included a large volume from the head up to the pelvic bones. For the lower body, an additional two scans were performed for the knees and foot regions. Only the first registration of the knee was applied, and averaging was not performed. If necessary, the position of the patient was corrected, and the scanning process was repeated.

In the case of a VMAT modality, CBCT registration was performed in the first treatment isocenter (head area) with translational shift applied to the current position of the patient. Next, several scans corresponding to the planned isocenters were carried out one after the other without application of registration results. The distance between isocenters was strictly controlled; the movement from one isocenter to another was carried out exclusively by moving the treatment couch in the longitudinal direction. Treatment began only after visualization of all planned positions was finished. If the results of image registration were unsatisfactory with respect to PTV or OAR positioning, the patient setup was manually adjusted, and all scans were repeated.

### Overall Assessment of Treatment Delivery Accuracy

For the VMAT treatment, several CBCTs corresponding to the planned isocenter positions were available. The translational shifts obtained during pretreatment visualization were applied to all CBCTs. Hounsfield unit to electron density (HU to ED) conversions were applied to the CBCT images in accordance with the predefined HU to ED calibration curves for each CBCT scanning protocol and the size of the patient. As a result, the impact of scatter contamination during CBCT image acquisition to HU was normalized, and original CBCTs were transformed into ED series (electron density series). Head, chest, abdomen, pelvis, and legs ED series were used to construct a single combined CBCT series of the full body. The combined CBCT series were masked with the planning CT to assure full image coverage at the FOV and generate the full CBCT if needed.

For TomoTherapy treatment, it is possible to select a scanning area with the required length from head to pelvis. However, the TomoTherapy MVCT scanner has an FOV of 40 cm, so the area outside the MVCT imaging diameter was masked with the planning CT to obtain full MVCT.

One third of the patients were scanned outside the FOV of the arms. Full MVCTs and CBCTs were used as primary series during deformable image registration (DIR), while the planning CTs were the secondary series. In this manner, the planning CTs were deformed to match the geometry of daily images, and synthetic CTs were obtained.

**Figure 2:**
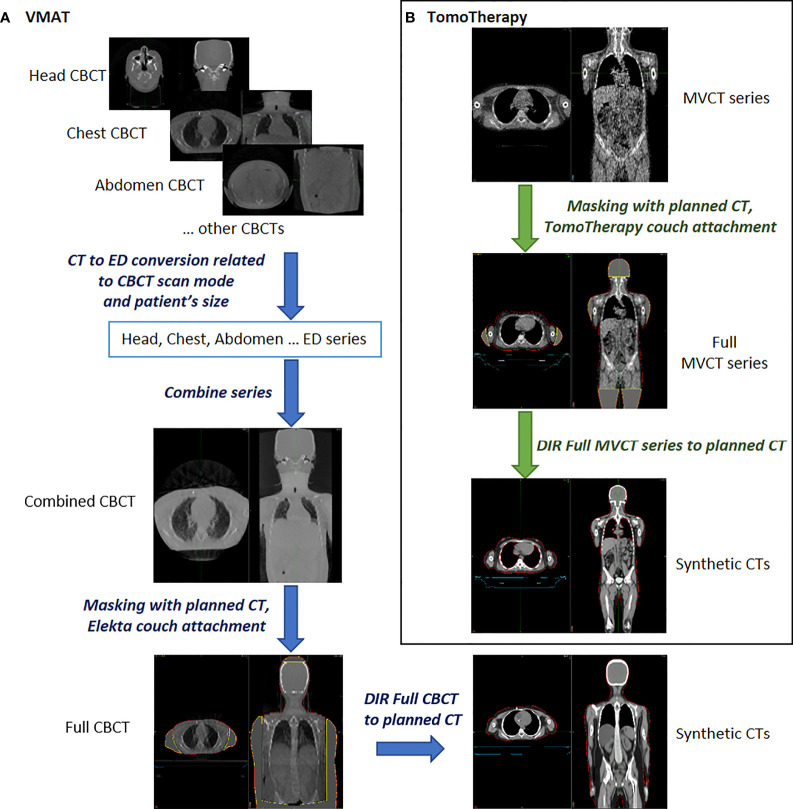
Overview of the data preprocessing for synthetic CT creation using predeveloped workflows in MIM Maestro^™^ software. **(A)** for VMAT **(B)** for TomoTherapy.

The procedures for processing, contouring, registering and deforming images were carried out using automated workflows of the MIM Maestro software. An overview of the data preprocessing is presented in **Figure 2**.

Synthetic CTs and original DICOM RT plan files were used to recalculate daily fractional dose. Dose calculations were performed using Monaco 5.11 for the VMAT plans and the MIM SureCalc^®^ MonteCarlo Plan verification module for the TomoTherapy plans. The Monte Carlo algorithm with an uncertainty of 1% was used in both cases.

The delivered doses (the sum of doses for all fractions) were compared to the planned dose by analyzing the dose-volume histograms (DVH) in terms of mean dose, near minimum (D_98min_) and near maximum (D_2max_) doses, 90% (D_90%_) and 95% (D_95%_) doses of an OAR structure, and the volumes of structures covered by 6 Gy (V6), 8 Gy (V8) and 10 Gy (V10). To assess dose delivery, the PTV was divided into several subvolumes. Both skeletal and regional PTV (head, chest, neck and shoulders, abdomen) dose variations were considered.

Overall assessment of the OC-TBI delivery accuracy was performed for each individual treatment fraction for 60 patients, 30 of whom were treated with VMAT and 30 with TomoTherapy, and for patients of different heights [≤130 cm (small) versus >130 cm (large)].

### Statistical Analysis

Statistical analysis was performed using IBM SPSS Statistics (USA) software. Two hundred and nine patients who received OC-TBI and underwent allogenic HSCT were included in the final analysis. Toxicity difference between 157 patients treated with TomoTherapy and 52 patients treated with VMAT was assessed with a chi-square test for independence. Comparison of planned doses for above patients groups and also the statistical analysis of delivered dose for 30 VMAT and 30 TomoTherapy patients was performed using unpaired two-sample t-tests at the 5% significance level. The normality of quantitative data was analyzed using Shapiro–Wilk test. To test the hypothesis of equality of variances we used F-test of equality of variances. The graphs of percentage difference between delivered (sum of all fractions) and planned doses were presented as a Box plot.

## Results

### Comparison of the OC-TBI Treatment Planning Results for Two Groups of Patients Treated by TomoTherapy and VMAT

A comparison of the DVH data for the OC-TBI treatment plans of the two patient groups (TomoTherapy and VMAT) who received standardized treatment is presented in **Tables 3** and **4**.

**Table 3:** OC-TBI treatment plan comparison for the two groups of patients who received standardized treatment.

Structure	Modality	D_2max_	D_90%_	D_95%_	D_98 min_	Dmean	HI
**PTV**	VMAT	13.31 ± 0.23	11.77 ± 0.14	11.39 ± 0.21	10.71 ± 0.33	12.29 ± 0.09	0.21 ± 0.05
	Tomo	12.86 ± 0.40	11.83 ± 0.10	11.65 ± 0.15	11.13 ± 0.33	12.09 ± 0.15	0.14 ± 0.04
	p	<0.01	<0.05	<0.01	<0.01	<0.01	<0.01
**Ribs**	VMAT	12.63 ± 0.22	10.68 ± 0.25	10.34 ± 0.26	9.98 ± 0.28	11.64 ± 0.20	0.42 ± 0.05
	Tomo	12.06 ± 0.52	10.62 ± 0.40	10.33 ± 0.40	10.02 ± 0.39	11.33 ± 0.44	0.36 ± 0.04
	p	<0.01	0.27	0.92	0.44	<0.01	<0.01

TomoTherapy group, n = 157 and VMAT group, n = 52. The following metrics are presented for the OC-TBI plans for target structures PTV and Ribs: D_2max_, D_90%_, D_95%_, D_98 min_, Dmean, V10 values and HI (the homogeneity index).

**Table 4:** OC-TBI treatment plan comparison for the two groups of patients who received standardized treatment.

Structure	Modality	Dmean	D_2max_	D_98 min_	V6	V8	V10
**Kidney_L**	VMAT	7.40 ± 0.28	9.80 ± 0.32	5.41 ± 0.35	83.84 ± 7.76	34.60 ± 7.01	1.78 ± 1.50
	Tomo	7.64 ± 0.34	11.12 ± 0.71	5.85 ± 0.57	83.78 ± 11.05	36.11 ± 7.70	10.22 ± 5.50
	p	<0.01	<0.01	<0.01	0.97	0.26	<0.01
**Kidney_R**	VMAT	7.48 ± 0.25	9.86 ± 0.31	5.54 ± 0.34	86.43 ± 7.11	36.43 ± 6.52	1.99 ± 1.61
	Tomo	7.64 ± 0.34	11.12 ± 0.69	5.84 ± 0.56	84.03 ± 10.96	35.95 ± 7.83	10.25 ± 5.30
	p	<0.01	<0.01	<0.01	0.08	0.79	<0.01
**Lung_L**	VMAT	7.72 ± 0.12	11.28 ± 0.29	5.32 ± 0.23	86.04 ± 4.17	38.81 ± 2.79	11.88 ± 1.98
	Tomo	7.85 ± 0.15	11.58 ± 0.39	6.12 ± 0.18	98.73 ± 2.35	37.55 ± 3.82	13.43 ± 3.62
	p	<0.01	<0.01	<0.01	<0.01	<0.05	<0.01
**Lung_R**	VMAT	7.67 ± 0.12	11.30 ± 0.26	5.19 ± 0.27	83.28 ± 4.86	38.45 ± 2.66	12.26 ± 2.04
	Tomo	7.80 ± 0.15	11.55 ± 0.34	6.11 ± 0.22	98.45 ± 2.91	36.52 ± 3.96	12.84 ± 3.23
	p	<0.01	<0.01	<0.01	<0.01	<0.01	0.17
**Lens_L**	VMAT	6.15 ± 0.43					
	Tomo	6.05 ± 0.74					
	p	1.34					
**Lens_R**	VMAT	6.33 ± 0.53					
	Tomo	5.99 ± 0.76					
	p	0.74					

TomoTherapy group, n = 157 and VMAT group, n = 52. For the OC-TBI plans, the metrics Dmean, D_2max_, D_98 min_, V6, V8, V10 values are presented for the organ at risk structures Lung_L Lung_R, Kidney_L and Kidney_R and the mean doses for the Lens_L and Lens_R structures.

**Figure 3:**
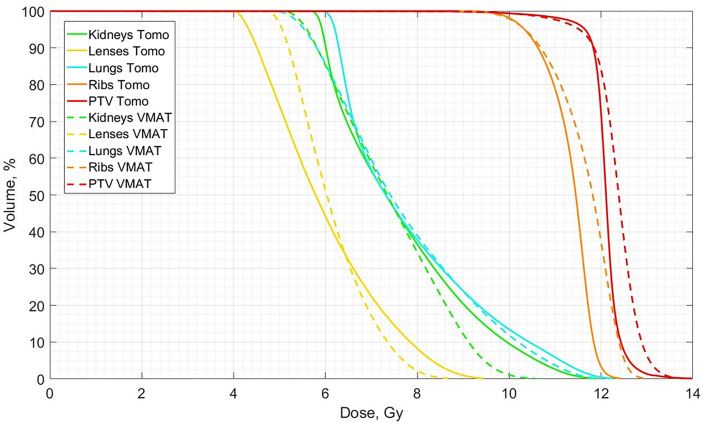
Comparison of averaged dose-volume histograms between the standardized OC-TBI for TomoTherapy (n = 157, Tomo, solid lines) and VMAT (n = 52, VMAT, dotted lines) plans.

A comparison of the averaged dose-volume histograms between the two groups of patients who received standardized OC-TBI with TomoTherapy (n = 157) and VMAT (n = 52) is shown in **Figure 3**.

### Individual Quality Assurance Procedures for OC-TBI Treatment Plans

For the VMAT treatment plans, the results of composite dose verification with applied angular sensitivity correction, the percentages of the points meeting the 2%/2 mm and 3%/3 mm Gamma criteria, were 92.1 ± 1.7% (s.d.) and 99.2 ± 1.6% (s.d.), respectively.

The TomoTherapy-based plans showed satisfactory results in dosimetry assessment. In 96% of the cases, the measured dose was within 3% of the calculated value from the TPS. Comparison of the exit detector data with a sinogram from the TPS indicated that the percentage of points meeting the 3%/3 mm Gamma criterion were 95.3 ± 1.9% (s.d.).

### Overall Assessment of Treatment Delivery Accuracy for VMAT- and TomoTherapy-Based OC-TBI

**Figure 4:**
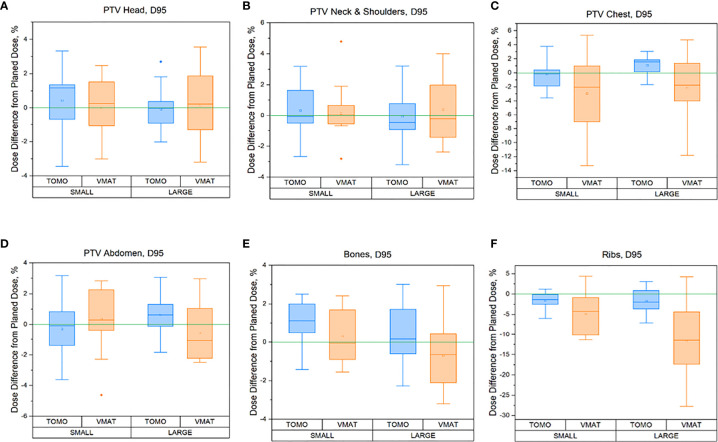
Regional percentage dose differences between delivered (sum of all fraction) and planned 95% (D95%) doses for the TomoTherapy and VMAT OC-TBI plans for the following six PTV subregions for small (height ≤130 cm, n = 30) and large patients (height >130 cm) patients: **(A)** skeleton (Bones), **(B)** PTV head, **(C)** PTV_Neck&Shoulders, **(D)** PTV_Chest, **(E)** PTV_Abdomen, and **(F)** Ribs.

The percentage dose difference between the delivered (sum of all fractions) and planned 95% dose (D95%) for the following six subregions of the PTV is shown in **Figure 4**: a) skeleton (Bones), b) PTV head, c) PTV_Neck&Shoulders, d) PTV_Chest, e) PTV_Abdomen, and f) Ribs. A comparison of the TomoTherapy and VMAT OC-TBI plans for patients with height ≤130 cm (small, n = 30) and height >130 cm (large, n = 30) is also presented.

A full data comparison between the delivered doses and planned dose to PTV subregions in terms of near maximum D_2max_, 90% (D_90%_), 95% (D_95%_), near minimum D_98 min_ of a structure, and mean dose for the TomoTherapy and VMAT OC-TBI modalities and for different patient heights [≤130 cm (small) and >130 cm (large)] are presented in **Supplementary Table 1**.

**Figure 5:**
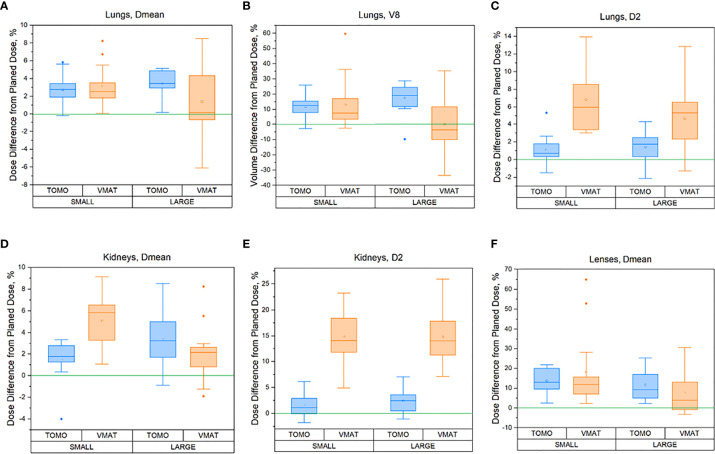
Percentage difference between delivered (sum of all fractions) and planned doses for the OARs for different patient heights [≤130 cm (small) and >130 cm (large)]: **(A)** Lungs in terms of Dmean, **(B)** Lungs in terms of V8, **(C)** Lungs in terms of D2max, **(D)** Kidneys in terms of Dmean, **(E)** Kidneys in terms of D2max, **(F)** Lens in terms of Dmean for TomoTherapy and VMAT OC-TBI plans.

The VMAT and TomoTherapy percentage dose differences for the OARs between the delivered (sum of all fractions) and planned dose are displayed in **Figure 5**.

A full data comparison between the delivered doses (sum of doses for all fractions) and planned doses to the OARs in terms of mean, near maximum D_2max_, and near minimum D_98 min_ dose of a structure and 90% volumes of structures covered by 6 Gy (V6), 8 Gy (V8), and 10 Gy (V10) for the TomoTherapy and VMAT OC-TBI modalities and for different patient heights [≤130 cm (small) and >130 cm (large)] are presented in **Supplementary Table 2**.

**Figure 6:**
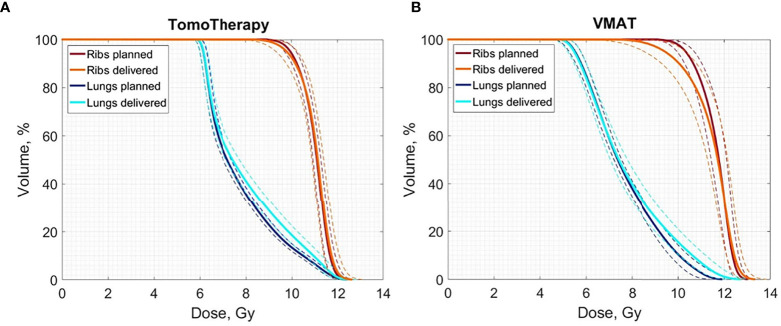
Averaged delivered (sum of all fractions) and planned Lungs and Ribs DVH for the 30 **(A)** TomoTherapy and **(B)** 30 VMAT OC-TBI plans. Blue line represents the resulting delivered lung dose, dark blue represents the original lung planned dose, and orange line represents the resulting delivered rib dose and dark red represents the original planned rib dose; dotted lines represent standard deviations.

The averaged planned and delivered DVHs for the lung and ribs for both TomoTherapy and VMAT OC-TBI are displayed in **Figure 6**. The blue line represents the resulting delivered lung dose, the dark blue line represents the original lung planned dose, the orange line represents the resulting delivered rib dose and the dark red line represents the original planned rib dose; the dotted lines represent the standard deviations.

### Toxicity Assessment

The results of acute toxicity during radiation therapy are presented in **Table 5**.

**Table 5:** The results of acute toxicity during radiation therapy in TomoTherapy and VMAT patients.

Toxicity criteria (RTOG)	TomoTherapy	Vmat	P-value
Number of pts	157	52	
Nausea and vomit			
Grade 0–1	104 (66%)	33 (63%)	0.71
Grade 2–3	54 (34%)	19 (37%)	
Headache			
Grade 0–1	103 (65%)	36 (69%)	0.63
Grade 2–3	54 (35%)	16 (31%)	
Parotitis			
No clinical symptoms	63 (51%)	27 (52%)	0.14
1 Grade clinical symptoms	94 (59%)	25 (49%)	
Enteritis			
No clinical symptoms	97 (62%)	30 (57%)	0.87
Grade 1	47 (30%)	17 (33%)	
Grade 2	13 (8%)	5 (10%)	

Subacute toxicity (IP) up to the 100th day after HSCT was not observed. The median follow up period was 2.3 years. The late toxicity from kidneys and lungs was not found during the follow up period.

## Discussion

### OC-TBI Clinical Implementation

The introduction of a standardized OC-TBI treatment significantly reduces the treatment preparation time and results in an overall more straightforward implementation of OC-TBI procedures in routine practice. This has enabled us to perform OC-TBI for three patients per week in a busy radiotherapy department setting, ensuring continuity of the radiation treatment course for patients, despite possible equipment technical malfunctions. A prerequisite for the successful implementation of OC-TBI is good communication and collaboration within the team of radiation therapists, hematologists, and medical physicists.

During pretreatment imaging, the user has access to various settings for the scanning protocols. In this case, the quality of the resulting image trades off with the speed of the scanning process and the imaging dose applied to the patient. We used fast scan protocols with bone registration for both CBCT and MVCT. The CBCT image feature has better soft tissue contrast and image resolution than MVCT, but separate scans are required for each isocenter position. MVCT does not have limitations related to the maximum length of the scan area. Zuro et al. (16) showed the advantage of whole-body imaging over partial body imaging in reducing overall patient positioning error.

Due to its technical features, the Elekta treatment unit used in VMAT treatment is less capable of irradiation of long targets than TomoTherapy. The development of the OC-TBI technique using VMAT required additional effort in treatment planning and also the physical and technical provision of quality assurance procedures. In our department, we use OC-TBI with VMAT as a backup modality to irradiate patients without potential interruption from technical issues. No patients are specifically selected for VMAT.

VMAT-based OC-TBI plans have a high number of Monitor Units. On average, one VMAT field accounts for 838 [670; 1,058] Monitor Units for the head or pelvis region and 1,760 [1,296; 2,276] MU for the chest or abdomen, which is caused by a high degree of plan modulation due to sparing of the organs at risk. Despite this fact, the results of a composite dose verification of the VMAT treatment plans appeared to be satisfactory. The requirement to use multiple overlapping fields in VMAT-based OC-TBI plans can lead to additional uncertainties regarding the actual delivered dose in the area of beam overlap. The feasibility and safety of the VMAT method had been previously demonstrated (24, 30, 31).

In the case of OC-TBI or TMLI, whole-body structures must be contoured. Automation of the contouring procedures is highly advantageous and ensures a reduction in the duration of the procedure. For VMAT, a significant number of additional structures is required for dose control in the area of beam overlap and junctions. Initially, we spent at least 1 h contouring structures for TomoTherapy (25 structures) and 2 h for VMAT (38 structures). After introducing semiautomated contouring methods, namely, preconfigured workflows, using MIM Maestro™ software (MIM Software Inc., Clevland, OH, USA), the duration of the contouring process was reduced up to 10–15 min for both modalities.

The time required for full calculation of the treatment plan using TomoTherapy 4.5 Non-VoLO™ Systems ranges from 2 to 5 h per patient, depending on the size of the patient. We used 200–250 iterations in the background without staff involvement. Together with the initial beamlet calculation, this took up to 4.5 h and then up to approximately 50 iterations with the participation of the planner. Template-based planning speeds up the process and reduces the dose calculation time involving staff up to 40 min.

The treatment planning speed depends not only on the size of the patient but also on the hardware configuration of the TPS. Using our HP 840 workstation with 256 GB of RAM required up to 4 h to calculate the full dose of VMAT-based OC-TBI. Using plan templates reduced the required time involving staff to approximately 2 h per plan.

The dose delivery time depends on patient height and is approximately the same for both modalities—on average 30 min (from 16 to 50 min)—but the total treatment time is different, approximately up to 60 min for TomoTherapy versus up to 90 min for VMAT. Applying surface scanning systems and recent advances in imaging equipment may reduce the total fraction time, making it practical for routine application (25).

### Comparison of the OC-TBI Treatment Planning Results Between TomoTherapy and VMAT

When applying the OC-TBI approach initially developed for TomoTherapy to the VMAT modality, we experienced challenges in meeting certain dose constraint criteria.

Meeting the acceptance criteria of V8 <40% for the lungs was only possible if another plan acceptance criterion related to the 6 Gy lung coverage was violated. Thus, the near minimum dose to the lungs is approximately 6 Gy for TomoTherapy and 5 Gy for the VMAT plans. The lung volume receiving a dose of 8 Gy did not exceed 40% for either case, but its mean dose differed slightly. The average dose to the kidneys was similar for TomoTherapy and the VMAT, while the volume of kidneys that received the near maximum dose was 13.1% lower for the VMAT plans due to the smoother achieved dose gradient.

Both methods showed acceptable quality in the PTV dose coverage while maintaining OAR sparing, but the mean PTV dose was 1.6% higher in the VMAT plans. The uniformity of PTV irradiation was superior for TomoTherapy, as evidenced by the lower HI values for TomoTherapy relative to VMAT.

### Overall Assessment of Treatment Delivery Accuracy for VMAT- and TomoTherapy-Based OC-TBI

Pretreatment visualizations (MVCT or CBCT) were used to correct patient position prior to treatment. However, the anatomical changes of the patient between fractions and positioning errors affect dose delivery during the treatment course. Accurate knowledge of the delivered dose to the OARs and to the target could prove advantageous in the future analysis of treatment outcomes.

Pediatric patients have a significant range in body size. We evaluated whether our OC-TBI methods provide sufficient plan robustness when applied to patients of varying sizes. Differences in the patient size did not result in significant differences for most parameters in either TomoTherapy- or VMAT-based OC-TBI.

However, differences between TomoTherapy and VMAT delivery were observed. In general, TomoTherapy-based OC-TBI treatment plans were more robust and less affected by variations in daily patient positioning (**Figure 6**, **Supplementary Table 2**). The percentage difference between planned and delivered doses (sum of all fractions) in the D_2max_ value for both the lungs and kidneys showed higher values for VMAT than for TomoTherapy. Thus, the D_2max_ percentage difference between the delivered (sum of all fractions) and planned dose in the right kidneys (large height group) was 2.4 [1.1; 3.7] % for TomoTherapy versus 15.1 [12.4; 17.8] % for VMAT plans. This might have been caused by the features of VMAT OC-TBI treatment plans, which had higher mean PTV doses and contained hotspots in the abdomen/pelvic junction area (**Figure 1**). Given that the planned D_2max_ dose in the kidneys was lower for VMAT than for TomoTherapy (9.8 versus 11.1 Gy), the absolute values of the delivered dose looked acceptable (11.3 vs. 11.4 Gy).

The parts of the lungs receiving doses in excess of 8 Gy included a high dose gradient and were susceptible to dose delivery variations (**Figure 6** **)**. These areas were characterized by an approximately 10% excess of the delivered dose relative to the planned dose for both TomoTherapy and VMAT. At the same time, the near minimum lung doses were less subject to changes. These results are consistent with the report of Zuro (16). Nevertheless, the mean delivered dose to the lungs increased by an average of less than 5.4% relative to the planned dose.

In relation to the mean dose to the PTV and its subregions, both modalities showed consistent results. The average percentage difference between delivered and planned mean doses to the PTV subregions was within 1% (**Supplementary Table 1**). The variation between planned and delivered values of D_95%_ coverage for the Head, Neck and Shoulders, Abdomen and Bones PTV subregions remained within 5% for both the TomoTherapy and VMAT modalities (**Figure 4**), which indicates the reliability of dose delivery to these regions.

**Figure 6** shows that TomoTherapy plans were more robust with respect to D_95%_ for rib dose coverage. We observed higher percentage variations in the Ribs D_95%_ for the VMAT plans [−11.5 (−15.7, −7.3) %] than for TomoTherapy [−1.7 (−3.3, −0.1) %] in the large height patient group. This could be caused by the steeper dose gradient in the chest area for the TomoTherapy planning dose distribution relative to VMAT.

Our method of estimating the delivered dose has limitations. We do not receive images during or after the treatment. If the patient changes his position during the treatment, we cannot consider this. An important advantage of OC-TBI is the ability to treat pediatric patients in the supine position, which is the most comfortable for the patient and provides good reproducibility of the patient setup and high accuracy of the dose delivery

### Conclusions and Future Direction

The study demonstrates that both the TomoTherapy and VMAT modalities are feasible, safe and provide acceptable toxicity in pediatric OC-TBI.

Our previous results also demonstrated that OC-TBI appears to be a promising technique for the treatment of pediatric patients (43, 44). Applying a standardization approach allowed us to homogeneously implement pediatric OC-TBI in routine clinical practice. OC-TBI is a technically complex, resource-intensive treatment modality, and its implementation requires automation and standardization at all stages of pretreatment preparation.

Despite of the detected planned and delivered dose difference between TomoTherapy and VMAT there were no significant differences in acute and subacute toxicity. The developed standardized OC-TBI with accurate dose delivery assessment may give the possibility to investigate the correlation between the delivered dose and the clinical outcomes. Automation of the pretreatment processes and application of fast semiautomatic planning or knowledge-based planning optimization solutions will help increase the availability of TBI/TMLI treatment techniques for more patients.

The accumulation of new clinical data and potential advantages of OAR sparing combined with possible target dose escalation could open new possibilities for the transition from OC-TBI to new, more targeted approaches for certain cohorts of pediatric patients.

## Data Availability Statement

The original contributions presented in the study are included in the article/**Supplementary Material**. Further inquiries can be directed to the corresponding author.

## Ethics Statement

The studies involving human participants were reviewed and approved by the Local Ethics Committee of the Dmitry Rogachev National Medical Research Center of Pediatric Hematology, Oncology and Immunology. Written informed consent to participate in this study was provided by the participants’ legal guardian/next of kin.

## Author Contributions

AAL, DT, DK, AOL, and AN contributed equally to this work. AAL, DT and AN designed the study, collected and analyzed data, and wrote the paper. AOL collected and analyzed data. DK collected, analyzed data and wrote the paper. MM and AC contributed to study design and reviewed the paper. OE contributed to data analysis and reviewed the paper. All authors listed have made a substantial, direct, and intellectual contribution to the work and approved it for publication.

## Conflict of Interest

The authors declare that the research was conducted in the absence of any commercial or financial relationships that could be construed as a potential conflict of interest.

## Publisher’s Note

All claims expressed in this article are solely those of the authors and do not necessarily represent those of their affiliated organizations, or those of the publisher, the editors and the reviewers. Any product that may be evaluated in this article, or claim that may be made by its manufacturer, is not guaranteed or endorsed by the publisher.

## References

[1] Carruthers SA Wallington MM. Total Body Irradiation and Pneumonitis Risk: A Review of Outcomes. Brit J Cancer (2004) 90(11):2080–4. doi: 10.1038/sj.bjc.6601751 PMC2409505 15150598

[2] Kelsey CR Horwitz ME Chino JP Craciunescu O Steffey B Folz RJ. Severe Pulmonary Toxicity After Myeloablative Conditioning Using Total Body Irradiation: An Assessment of Risk Factors. Int J Radiat Oncol Biol Phys (2011) 81(3):812–8. doi: 10.1016/j.ijrobp.2010.06.058 20932682

[3] Abugideiri M Nanda RH Butker C Zhang C Kim S Chiang KY. Factors Influencing Pulmonary Toxicity in Children Undergoing Allogeneic Hematopoietic Stem Cell Transplantation in the Setting of Total Body Irradiation-Based Myeloablative Conditioning. Int J Radiat Oncol Biol Phys (2016) 94(2):349–59. doi: 10.1016/j.ijrobp.2015.10.054 26853343

[4] Keane JT Fontenla DP Chui CS. Applications of IMAT to Total Body Radiation (TBI). Int J Radiat Oncol Biol Phys (2000) 48(3S1):239–9. doi: 10.1016/S0360-3016(00)80274-6

[5] Hussain A Villarreal-Barajas JE Dunscombe P Brown DW. Aperture Modulated, Translating Bed Total Body Irradiation. Med Phys (2011) 38(2):932–41. doi: 10.1118/1.3534196 21452729

[6] Hui SK Kapatoes J Fowler J Henderson D Olivera G Manon RR. Feasibility Study of Helical Tomotherapy for Total Body or Total Marrow Irradiation. Med Phys (2005) 32(10):3214–24. doi: 10.1118/1.2044428 16279075

[7] Wong JY Liu A Schultheiss T Popplewell L Stein A Rosenthal J. Targeted Total Marrow Irradiation Using Three-Dimensional Image-Guided Tomographic Intensity-Modulated Radiation Therapy: An Alternative to Standard Total Body Irradiation. Biol Blood Marrow Transplant (2006) 12(3):306–15. doi: 10.1016/j.bbmt.2005.10.026 16503500

[8] Hui SK Verneris MR Froelich J Dusenbery K Welsh JS. Multimodality Image Guided Total Marrow Irradiation and Verification of the Dose Delivered to the Lung, PTV, and Thoracic Bone in a Patient: A Case Study. Technol Cancer Res Treat (2009) 8(1):23–8. doi: 10.1177/153303460900800104 19166239

[9] Aydogan B Mundt AJ Roeske JC. Linac-Based Intensity Modulated Total Marrow Irradiation (IM-TMI). Technol Cancer Res Treat (2006) 5(5):513–9. doi: 10.1177/153303460600500508 16981794

[10] Wilkie JR Tiryaki H Smith BD Roeske JC Radosevich JA Aydogan B. Feasibility Study for Linac-Based Intensity Modulated Total Marrow Irradiation. Med Phys (2008) 35(12):5609–18. doi: 10.1118/1.2990779 19175118

[11] Ouyang L Folkerts M Zhang Y Hrycushko B Lamphier R Lee P. Volumetric Modulated Arc Therapy Based Total Body Irradiation: Workflow and Clinical Experience With an Indexed Rotational Immobilization System. Phys Imaging Radiat Oncol (2017) 4:22–5. doi: 10.1016/j.phro.2017.11.002

[12] Tas B Durmus IF Okumus A Uzel OE Gokce M Goksoy HS. Total-Body Irradiation Using Linac-Based Volumetric Modulated Arc Therapy: Its Clinical Accuracy, Feasibility and Reliability. Radiother Oncol (2018) 129(3):527–33. doi: 10.1016/j.radonc.2018.08.005 30172456

[13] Springer A Hammer J Winkler E Track C Huppert R Bohm A. Total Body Irradiation With Volumetric Modulated Arc Therapy: Dosimetric Data and First Clinical Experience. Radiat Oncol (2016) 11:46. doi: 10.1186/s13014-016-0625-7 27000180 PMC4802832

[14] Gruen A Ebell W Wlodarczyk W Neumann O Kuehl JS Stromberger C. Total Body Irradiation (TBI) Using Helical Tomotherapy in Children and Young Adults Undergoing Stem Cell Transplantation. Radiat Oncol (2013) 8:92. doi: 10.1186/1748-717X-8-92 23587349 PMC3653702

[15] Wong JY Filippi AR Scorsetti M Hui S Muren LP Mancosu P. Total Marrow and Total Lymphoid Irradiation in Bone Marrow Transplantation for Acute Leukaemia. Lancet Oncol (2020) 21(10):e477–87. doi: 10.1016/S1470-2045(20)30342-9 33002443

[16] Zuro D Vagge S Broggi S Agostinelli S Takahashi Y Brooks J. Multi-Institutional Evaluation of MVCT Guided Patient Registration and Dosimetric Precision in Total Marrow Irradiation: A Global Health Initiative by the International Consortium of Total Marrow Irradiation. Radiother Oncol (2019) 141:275–82. doi: 10.1016/j.radonc.2019.07.010 PMC8819916 31421913

[17] Stein A Palmer J Tsai NC Al Malki MM Aldoss I Ali H. Phase I Trial of Total Marrow and Lymphoid Irradiation Transplantation Conditioning in Patients With Relapsed/Refractory Acute Leukemia. Biol Blood Marrow Transplant (2017) 23(4):618–24. doi: 10.1016/j.bbmt.2017.01.067 PMC5382014 28087456

[18] Kim JH Stein A Tsai N Schultheiss TE Palmer J Liu A. Extramedullary Relapse Following Total Marrow and Lymphoid Irradiation in Patients Undergoing Allogeneic Hematopoietic Cell Transplantation. Int J Radiat Oncol Biol Phys (2014) 89(1):75–81. doi: 10.1016/j.ijrobp.2014.01.036 24725691

[19] Hoeben BAW Pazos M Albert MH Seravalli E Bosman ME Losert C. Towards Homogenization of Total Body Irradiation Practices in Pediatric Patients Across SIOPE Affiliated Centers. A Survey by the SIOPE Radiation Oncology Working Group. Radiother Oncol (2021) 155:113–9. doi: 10.1016/j.radonc.2020.10.032 33137397

[20] Rassiah P Esiashvili N Olch AJ Hua CH Ulin K Molineu A. Practice Patterns of Pediatric Total Body Irradiation Techniques: A Children’s Oncology Group Survey. Int J Radiat Oncol Biol Phys (2021) 111(5):1155–64. doi: 10.1016/j.ijrobp.2021.07.1715 PMC11977860 34352289

[21] Esiashvili N Lu X Ulin K Laurie F Kessel S Kalapurakal JA. Higher Reported Lung Dose Received During Total Body Irradiation for Allogeneic Hematopoietic Stem Cell Transplantation in Children With Acute Lymphoblastic Leukemia Is Associated With Inferior Survival: A Report From the Children’s Oncology Group. Int J Radiat Oncol Biol Phys (2019) 104(3):513–21. doi: 10.1016/j.ijrobp.2019.02.034 PMC6548591 30807822

[22] Frassoni F Scarpati D Bacigalupo A Vitale V Corvo R Miceli S. The Effect of Total Body Irradiation Dose and Chronic Graft-Versus-Host Disease on Leukaemic Relapse After Allogeneic Bone Marrow Transplantation. Br J Haematol (1989) 73(2):211–6. doi: 10.1111/j.1365-2141.1989.tb00254.x 2684259

[23] Scarpati D Frassoni F Vitale V Corvo R Franzone P Barra S. Total Body Irradiation in Acute Myeloid Leukemia and Chronic Myelogenous Leukemia: Influence of Dose and Dose-Rate on Leukemia Relapse. Int J Radiat Oncol Biol Phys (1989) 17(3):547–52. doi: 10.1016/0360-3016(89)90105-3 2674077

[24] Cox JD Stetz J Pajak TF. Toxicity Criteria of the Radiation Therapy Oncology Group (RTOG) and the European Organization for Research and Treatment of Cancer (EORTC). Int J Radiat Oncol Biol Phys (1995) 31(5):1341–46. doi: 10.1016/0360-3016(95)00060-C 7713792

[25] Haraldsson A Engellau J Lenhoff S Engelholm S Bäck S Engström PE. Implementing Safe and Robust Total Marrow Irradiation Using Helical Tomotherapy - A Practical Guide. Phys Med (2019) 60:162–7. doi: 10.1016/j.ejmp.2019.03.032 31000078

[26] Takahashi Y Verneris MR Dusenbery KE Wilke CT Storme G Weisdorf DJ. Peripheral Dose Heterogeneity Due to the Thread Effect in Total Marrow Irradiation With Helical Tomotherapy. Int J Radiat Oncol Biol Phys (2013) 87(4):832–9. doi: 10.1016/j.ijrobp.2013.07.017 PMC3805769 24011657

[27] Chen M Chen Y Chen Q Lu W. Theoretical Analysis of the Thread Effect in Helical TomoTherapy. Med Phys (2011) 38(11):5945–60. doi: 10.1118/1.3644842 22047359

[28] Usui K Isobe A Hara N Shikama N Sasai K Ogawa K. Appropriate Treatment Planning Method for Field Joint Dose in Total Body Irradiation Using Helical Tomotherapy. Med Dosim (2019) 44(4):344–53. doi: 10.1016/j.meddos.2018.12.003 30598391

[29] Loginova AA Tovmasyan DA Chernyaev AP Varzar’ SM Kobyzeva DA Nechesnyuk AV. Field Junction Technique for Helical Tomotherapy-Based Total Body Irradiation. Med Radiol Radiat Saf (2018) 63(2):55–61. doi: 10.12737/article_5ac622371650f7.48983677

[30] Aydogan B Yeginer M Kavak GO Fan J Radosevich JA Gwe-Ya K. Total Marrow Irradiation With RapidArc Volumetric Arc Therapy. Int J Radiat Oncol Biol Phys (2011) 81(2):592–9. doi: 10.1016/j.ijrobp.2010.11.035 21345619

[31] Mancosu P Navarria P Reggiori G Cozzi L Fogliata A Gaudino A. *In-Vivo* Dosimetry With Gafchromic Films for Multi-Isocentric VMAT Irradiation of Total Marrow Lymph-Nodes: A Feasibility Study. Radiother Oncol (2015) 10:86. doi: 10.1186/s13014-015-0391-y PMC4397694 25881084

[32] Fogliata A Cozzi L Clivio A Ibatici A Mancosu P Navarria P. Preclinical Assessment of Volumetric Modulated Arc Therapy for Total Marrow Irradiation. Int J Radiat Oncol Biol Phys (2011) 80(2):626–36. doi: 10.1016/j.ijrobp.2010.11.028 21277109

[33] Han C Schultheisss TE Wong JY. Dosimetric Study of Volumetric Modulated Arc Therapy Fields for Total Marrow Irradiation. Radiother Oncol (2012) 102(2):315–20. doi: 10.1016/j.radonc.2011.06.005 21724284

[34] Symons K Morrison C Parry J Woodings S Zissiadis Y. Volumetric Modulated Arc Therapy for Total Body Irradiation: A Feasibility Study Using Pinnacle3 Treatment Planning System and Elekta Agility™ Linac. J Appl Clin Med Phys (2018) 19(2):103–10. doi: 10.1002/acm2.12257 PMC5849856 29368389

[35] Nalichowski A Eagle DG Burmeister J. Dosimetric Evaluation of Total Marrow Irradiation Using 2 Different Planning Systems. Med Dosim (2016) 41(3):230–35. doi: 10.1016/j.meddos.2016.06.001 27372384

[36] Mancosu P Navarria P Castagna L Roggio A Pellegrini C Reggiori G. Anatomy Driven Optimization Strategy for Total Marrow Irradiation With a Volumetric Modulated Arc Therapy Technique. J Appl Clin Med Phys (2012) 13(1):138–47. doi: 10.1120/jacmp.v13i1.3653 PMC5716136 22231216

[37] Mancosu P Navarria P Castagna L Reggiori G Stravato A Gaudino A. Plan Robustness in Field Junction Region From Arcs With Different Patient Orientation in Total Marrow Irradiation With VMAT. Phys Med (2015) 31(7):677–82. doi: 10.1016/j.ejmp.2015.05.012 26068115

[38] Wang L Qiu G Yu J Zhang Q Man L Chen L. Effect of Auto Flash Margin on Superficial Dose in Breast Conserving Radiotherapy for Breast Cancer. J Appl Clin Med Phys (2021) 22(6):60–70. doi: 10.1002/acm2.13287 PMC8200433 34028963

[39] Tovmasian DA Loginova AA Chernyaev AP Nechesnyuk AV. Non-Standard Use of TomoTherapy Exit Imaging Detectors for Quality Assurance Procedures. Moscow Univ Phys Bull+ (2021) 76(6):470–76. doi: 10.3103/S0027134921060096

[40] Low DA Harms WB Mutic S Purdy JA. A Technique for the Quantitative Evaluation of Dose Distributions. Med Phys (1998) 25(5):656–61. doi: 10.1118/1.598248 9608475

[41] Miften M Olch A Mihailidis D Moran J Pawlicki T Molineu A. Tolerance Limits and Methodologies for IMRT Measurement-Based Verification QA: Recommendations of AAPM Task Group No. 218. Med Phys (2018) 45(4):e53–83. doi: 10.1002/mp.12810 29443390

[42] Loginova AA Tovmasian DA Kokoncev DA Varzar SM Chernyaev AP. Angular Dependence Investigation of the MatriXX Detector Array for Dosimetric Verification of Treatment Plans With Intensity Modulation. Moscow Univ Phys Bull+ (2021) 76(5):384–91. doi: 10.3103/S0027134921050118

[43] Kobyzeva D Shelikhova L Shekhovtsova Z Khismatullina R Ilushina M Loginova A. Total Body Irradiation Among Recipients of Tcrαβ/CD19-Depleted Grafts in a Cohort of Children With Hematologic Malignances: Single Center Experience. Blood (2020) 136:2–2. doi: 10.1182/blood-2020-139282

[44] Dunaikina M Zhekhovtsova Z Shelikhova L Glushkova S Nikolaev R Blagov S. Safety and Efficacy of the Low-Dose Memory (CD45RA-Depleted) Donor Lymphocyte Infusion in Recipients of αβ T Cell-Depleted Haploidentical Grafts: Results of a Prospective Randomized Trial in High-Risk Childhood Leukemia. Bone Marrow Transpl (2021) 56(7):1614–24. doi: 10.1038/s41409-021-01232-x 33594278

