# Supplementary material for: Optimized Conformal Total Body Irradiation methods with Helical TomoTherapy and Elekta VMAT: Implementation, Imaging, Planning and Dose Delivery for Pediatric Patients
Source: Front Oncol. 2022 Mar 10;12:785917. doi: 10.3389/fonc.2022.785917 (PMC8960917; doi:10.3389/fonc.2022.785917)
Supplement: Supplementary file 1 [file Table_1.docx]

**Supplement Table 1**. Full data comparison between the delivered doses (the sum of doses for all fractions) and planned dose to PTV subregions (Bones, PTV head, PTV_Neck&Shoulders, PTV_Chest, PTV_Abdomen, Ribs) in terms of D2_max_, 90% D90, D95, D98_min_, D mean, for TomoTherapy and VMAT OC-TBI modalities and the different height of patients ≤130 cm (small) and >130cm (large). The statistical comparison of the TomoTherapy and VMAT modalities was performed using unpaired two-sample t-tests with the significance level of 5%

| **Structure** | **Size** | **Modality** | **D _2 max_** | **D 90** | **D 95** | **D _98 min_** | **D mean** | **HI** |
| --- | --- | --- | --- | --- | --- | --- | --- | --- |
| **PTV Head** | small | VMAT | 0.4 [-0.4, 1.2] | 0.1 [-0.7, 0.9] | 0 [-0.9, 0.9] | -0.2 [-1.6, 1.2] | 0 [-0.3, 0.3] | 38.1 [17.8, 58.4] |
|  |  | Tomo | 0.2 [-0.7, 1.1] | 0.3 [-0.7, 1.3] | 0.3 [-0.7, 1.3] | -0.5 [-1.6, 0.6] | -0.2 [-0.3, -0.1] | 45.7 [29.2, 62.2] |
|  |  | p | 0.781 | 0.773 | 0.685 | 0.694 | 0.168 | 0.574 |
|  | large | VMAT | 0.5 [-0.4, 1.4] | 0.6 [-0.2, 1.4] | 1.4 [-0.8, 3.6] | 3.3 [-0.8, 7.4] | 0.3 [0.1, 0.5] | 33.4 [15.2, 51.6] |
|  |  | Tomo | 0.8 [0.4, 1.2] | 0.1 [-0.6, 0.8] | -0.1 [-0.8, 0.6] | -2.1 [-3.1, -1.1] | 0.1 [-0.1, 0.3] | 4.9 [1.1, 8.7] |
|  |  | p | 0.431 | 0.496 | 0.296 | 0.034 | 0.001 | 0.595 |
| **PTV Neck and Shoulders** | small | VMAT | 1.2 [0.5, 1.9] | 0 [-0.5, 0.5] | 0.1 [-0.9, 1.1] | 0.1 [-2.1, 2.3] | 0.1 [-0.2, 0.4] | 4.9 [-4.1, 13.9] |
|  |  | Tomo | -0.2 [-1.2, 0.8] | 0.1 [-0.9, 1.1] | 0.3 [-0.7, 1.3] | 0.8 [-0.4, 2] | 0.2 [0.1, 0.3] | 4.3 [-7, 15.6] |
|  |  | p | 0.034 | 0.793 | 0.722 | 0.598 | 0.575 | 0.934 |
|  | large | VMAT | 1.7 [0.7, 2.7] | 0.9 [-0.3, 2.1] | 2.7 [-0.1, 5.5] | 5.4 [-0.2, 11] | 0.8 [0.3, 1.3] | 11.1 [-1.4, 23.6] |
|  |  | Tomo | -0.2 [-0.8, 0.4] | -0.2 [-1.1, 0.7] | -0.1 [-1, 0.8] | 0 [-0.8, 0.8] | -0.2 [-0.4, 0] | 4.3 [-2.5, 11.1] |
|  |  | p | 0.004 | 0.288 | 0.233 | 0.226 | 0.004 | 0.114 |
| **PTV Chest** | small | VMAT | 3 [1.8, 4.2] | -1.5 [-3.4, 0.4] | -2.9 [-5.8, 0] | -6 [-10.5, -1.5] | 0.4 [-0.2, 1] | 23.5 [14.3, 32.7] |
|  |  | Tomo | 0.6 [-0.2, 1.4] | 0.2 [-0.7, 1.1] | -0.2 [-1.5, 1.1] | -1.3 [-3, 0.4] | 0 [-0.2, 0.2] | 5.9 [2.8, 9] |
|  |  | p | 0.004 | 0.124 | 0.028 | 0.068 | 0.180 | 0.002 |
|  | large | VMAT | 4.1 [3, 5.2] | -0.5 [-2.2, 1.2] | -0.7 [-3.7, 2.3] | -3.4 [-8.1, 1.3] | 0.9 [0.4, 1.4] | 30.9 [20.6, 41.2] |
|  |  | Tomo | 0.6 [-0.1, 1.3] | 0.7 [0.1, 1.3] | 1.1 [0.4, 1.8] | 0.8 [-0.7, 2.3] | 0.1 [-0.1, 0.3] | 4.9 [1.1, 8.7] |
|  |  | p | <0.001 | 0.137 | 0.145 | 0.051 | 0.931 | <0.001 |
| **PTV Abdomen** | small | VMAT | 2.9 [2.1, 3.7] | 0.5 [-0.4, 1.4] | 0.4 [-0.7, 1.5] | -1.4 [-3.6, 0.8] | 0.6 [0.1, 1.1] | 21 [7.9, 34.1] |
|  |  | Tomo | -0.1 [-1.2, 1] | 0 [-1.1, 1.1] | -0.3 [-1.4, 0.8] | -2.4 [-5, 0.2] | -0.1 [-0.3, 0.1] | 19.5 [13, 26] |
|  |  | p | <0.001 | 0.468 | 0.417 | 0.593 | 0.016 | 0.839 |
|  | large | VMAT | 1.7 [0.6, 2.8] | 0.3 [-0.4, 1] | 1.2 [-0.3, 2.7] | 2.3 [-0.5, 5.1] | 0.4 [0.1, 0.7] | 33.5 [22.2, 44.8] |
|  |  | Tomo | 0.2 [-0.6, 1] | 0.6 [-0.2, 1.4] | 0.7 [0, 1.4] | 0.5 [-0.3, 1.3] | -0.1 [-0.3, 0.1] | 13.6 [7.6, 19.6] |
|  |  | p | 0.030 | 0.193 | 0.510 | 0.564 | 0.081 | 0.001 |
| **Ribs** | small | VMAT | 1.5 [0.7, 2.3] | -3.1 [-5.4, -0.8] | -4.8 [-7.5, -2.1] | -6.9 [-10.1, -3.7] | -0.5 [-1.5, 0.5] | 31.8 [20.8, 42.8] |
|  |  | Tomo | 0.7 [-0.3, 1.7] | -0.7 [-1.6, 0.2] | -1.5 [-2.7, -0.3] | -2.6 [-4.1, -1.1] | 0 [-0.4, 0.4] | 14.8 [8.2, 21.4] |
|  |  | p | 0.227 | 0.074 | 0.039 | 0.024 | 0.441 | 0.014 |
|  | large | VMAT | 1.6 [0.9, 2.3] | -7.5 [-10.8, -4.2] | -11.5 [-15.7, -7.3] | -15.9 [-20.9, -10.9] | -1.6 [-3, -0.2] | 52.7 [37.8, 67.6] |
|  |  | Tomo | 1.1 [0.2, 2] | -0.6 [-1.9, 0.7] | -1.7 [-3.3, -0.1] | -3.3 [-5.1, -1.5] | 0.4 [0, 0.8] | 22 [15.8, 28.2] |
|  |  | p | 0.337 | <0.001 | <0.001 | <0.001 | 0.013 | <0.001 |
| **Bones** | small | VMAT | 2.1 [1.5, 2.7] | 0.2 [-0.3, 0.7] | 0 [-0.8, 0.8] | -0.2 [-2.8, 2.4] | 0.3 [0, 0.6] | 24.5 [6.9, 42.1] |
|  |  | Tomo | 0.9 [0.2, 1.6] | 1.1 [0.5, 1.7] | 1.1 [0.5, 1.7] | 1 [0.2, 1.8] | 0.3 [0.2, 0.4] | 12.5 [3.9, 21.1] |
|  |  | p | 0.018 | 0.05 | 0.038 | 0.412 | 0.974 | 0.246 |
|  | large | VMAT | 1.2 [0.4, 2] | -0.3 [-1.1, 0.5] | -0.8 [-1.7, 0.1] | -2.8 [-4.6, -1] | 0.2 [-0.1, 0.5] | 27.6 [15.1, 40.1] |
|  |  | Tomo | -0.1 [-1, 0.8] | 0.2 [-0.7, 1.1] | 0.3 [-0.5, 1.1] | 0.5 [-0.2, 1.2] | 0.3 [0.2, 0.4] | 8.5 [-3.2, 20.2] |
|  |  | p | 0.037 | 0.378 | 0.082 | 0.003 | 0.556 | 0.035 |

**Supplement Table 2** Full data comparison between the delivered doses (the sum of doses for all fractions) and planned dose to OARs in terms of a structure’s D mean, D2_max_, D98_min_, V6, V8,V10 for TomoTherapy and VMAT OC-TBI modalities and the different height of patients ≤130 cm (small) and >130cm (large). The statistical comparison of the TomoTherapy and VMAT modalities was performed using unpaired two-sample t-tests with the significance level of 5%

| **Structure** | **Size** | **Modality** | **D mean** | **D 2 max** | **D 98 min** | **V 6** | **V 8** |
| --- | --- | --- | --- | --- | --- | --- | --- |
| **Kidney L** | small | VMAT | 5.1 [3.7, 6.5] | 14.9 [12, 17.8] | 1.1 [0, 2.2] | 2.5 [-0.4, 5.4] | 83.1 [-36.7, 202.9] |
|  |  | Tomo | 1.5 [0.5, 2.5] | 1.6 [0.4, 2.8] | 0.8 [-0.2, 1.8] | 1.3 [-1.3, 3.9] | 4.6 [0.6, 8.6] |
|  |  | p | <0.001 | <0.001 | 0.643 | 0.58 | 0.009 |
|  | large | VMAT | 2.1 [0.9, 3.3] | 14.8 [12.1, 17.5] | -1.3 [-2.5, -0.1] | -2.7 [-4.3, -1.1] | 4.5 [-1.4, 10.4] |
|  |  | Tomo | 3.3 [2.1, 4.5] | 2.4 [1.3, 3.5] | 0.7 [0, 1.4] | 3.5 [1.3, 5.7] | 14.6 [7, 22.2] |
|  |  | p | 0.139 | <0.001 | 0.009 | <0.001 | 0.007 |
| **Kidney R** | small | VMAT | 4.9 [3.2, 6.6] | 15.6 [12.1, 19.1] | 0.9 [-0.6, 2.4] | 2.3 [-1.5, 6.1] | 165.3 [-123, 453.6] |
|  |  | Tomo | 2.1 [1.1, 3.1] | 2.1 [1, 3.2] | 0.9 [-0.1, 1.9] | 2.2 [-0.7, 5.1] | 6.6 [0.9, 12.3] |
|  |  | p | 0.012 | <0.001 | 0.977 | 0.981 | 0.302 |
|  | large | VMAT | 2.1 [1.1, 3.1] | 15.1 [12.4, 17.8] | -1.7 [-3.3, -0.1] | -2.9 [-4.9, -0.9] | 2 [-3.7, 7.7] |
|  |  | Tomo | 2.9 [1.7, 4.1] | 2.4 [1.1, 3.7] | 0.7 [0, 1.4] | 3.2 [0.3, 6.1] | 13.7 [4.6, 22.8] |
|  |  | p | 0.282 | <0.001 | 0.011 | 0.002 | 0.042 |
| **Lung L** | small | VMAT | 3.1 [1.9, 4.3] | 6.9 [4.9, 8.9] | 0 [-0.6, 0.6] | -0.2 [-1, 0.6] | 13 [3.9, 22.1] |
|  |  | Tomo | 2.7 [1.8, 4.4] | 1.1 [0.1, 2.1] | 0.2 [-0.6, 1] | -0.4 [-1.1, 0.5] | 12 [7.7, 21.7] |
|  |  | p | 0.605 | <0.001 | 0.692 | 0.715 | 0.843 |
|  | large | VMAT | 1.4 [-0.6, 3.4] | 4.7 [3, 6.4] | -1 [-2.5, 0.5] | -2.2 [-5.3, 0.9] | 0.2 [-8.8, 9.2] |
|  |  | Tomo | 3.5 [2.8, 4.2] | 1.5 [0.7, 2.3] | 0.2 [-0.4, 0.8] | -0.2 [-0.9, 0.5] | 17.4 [12.6, 22.2] |
|  |  | p | 0.061 | 0.002 | 0.139 | 0.211 | 0.003 |
| **Lung R** | small | VMAT | 2.2 [1, 3.4] | 4.8 [3.2, 6.4] | -0.6 [-1.3, 0.1] | -1.3 [-3.1, 0.5] | 6.9 [-1.4, 15.2] |
|  |  | Tomo | 2.2 [1.3, 3.8] | 0.7 [-0.5, 1.9] | -0.2 [-1, 1.1] | -1 [-2, 0.7] | 10 [5.2, 19.5] |
|  |  | p | 0.928 | <0.001 | 0.467 | 0.812 | 0.515 |
|  | large | VMAT | 1.3 [-0.8, 3.4] | 6.2 [4, 8.4] | -0.6 [-1.9, 0.7] | -3.2 [-6.3, -0.1] | 0.5 [-7.7, 8.7] |
|  |  | Tomo | 2.9 [2.2, 3.6] | 1.6 [0.6, 2.6] | 0.3 [-0.5, 1.1] | -0.3 [-1.8, 1.2] | 14.7 [10.1, 19.3] |
|  |  | p | 0.144 | <0.001 | 0.268 | 0.110 | 0.006 |
| **Lens L** | small | VMAT | 18.2 [8.2, 28.2] |  |  |  |  |
|  |  | Tomo | 13.7 [10.6, 16.8] |  |  |  |  |
|  |  | p | 0.396 |  |  |  |  |
|  | large | VMAT | 7.6 [2.3, 12.9] |  |  |  |  |
|  |  | Tomo | 11.6 [8, 15.2] |  |  |  |  |
|  |  | p | 0.219 |  |  |  |  |
| **Lens R** | small | VMAT | 17.3 [7.8, 26.8] |  |  |  |  |
|  |  | Tomo | 12.6 [9.1, 16.1] |  |  |  |  |
|  |  | p | 0.362 |  |  |  |  |
|  | large | VMAT | 6.1 [0.8, 11.4] |  |  |  |  |
|  |  | Tomo | 9.8 [6.3, 13.3] |  |  |  |  |
|  |  | p | 0.267 |  |  |  |  |
